# Plasma cytokine profiles in breast cancer patients and their association with therapeutic response in Peru: a prospective cohort study

**DOI:** 10.3389/fimmu.2026.1771790

**Published:** 2026-06-09

**Authors:** Jose M. Vela-Ruiz, Zaida Morante, Yomali Ferreyra, Marco A. Galvez-Villanueva, Fernando Valencia, J. Jhanina Campos-Tineo, Mariana Callapiña De Paz, Ariana Alessandra Córdova-Salazar, Pool Marcos-Carbajal, Joan M. Moreno Lujan, Andy R. Pantoja Lazaro, Laura G. Escobar Caipo, Gustavo A. Flores Trujillo, Teresa N. Cusma Quintana, Jhony A. De La Cruz-Vargas, Henry L. Gomez, Alonso Soto

**Affiliations:** 1 Instituto de Investigaciones en Ciencias Biomédicas, Universidad Ricardo Palma, Lima, Peru; 2 Hospital Emergencias de Villa El Salvador, Lima, Peru; 3 Instituto Nacional de Enfermedades Neoplásicas - INEN, Lima, Peru; 4 Grupo de Estudios Clínicos Oncológicos del Peru - GECOPERU, Lima, Peru; 5 Health Innovation Laboratory, Institute of Tropical Medicine “Alexander von Humboldt,” Universidad Peruana Cayetano Heredia, Lima, Peru; 6 Instituto Regional de Enfermedades Neoplásicas del Norte - IREN Norte, Trujillo, Peru; 7 Universidad Católica de Santa María, Arequipa, Peru; 8 Instituto Regional de Enfermedades Neoplásicas del Sur - IREN Sur, Arequipa, Peru; 9 Universidad San Martin de Porres, Centro de Investigacion en Farmacología y Medicina Tradicional, Lima, Peru; 10 Universidad Nacional de Ucayali, Facultad de Medicina Humana, Pucallpa, Peru; 11 Oncosalud, Auna, Lima, Peru; 12 Universidad Peruana Cayetano Heredia, Lima, Peru

**Keywords:** breast cancer, cytokines, molecular subtypes, plasma biomarkers, treatment response

## Abstract

**Background:**

Breast cancer (BC) is the most common malignant neoplasm in women worldwide and in Peru. Beyond hormonal and genetic factors, cytokines play a key role in tumor aggressiveness and therapeutic resistance. However, evidence on circulating cytokine profiles in Latin American populations is limited.

**Objective:**

We aimed to characterize the plasma cytokine profile in Peruvian women with BC and evaluate its association with molecular subtypes and treatment response.

**Materials and methods:**

A prospective cohort study was conducted. We included 88 BC patients with clinical stage II–III, who were diagnosed at three different cancer centers in Peru: Instituto Nacional de Enfermedades Neoplásicas (INEN, Lima-Peru), Instituto Regional de Enfermedades Neoplásicas del Norte (IREN-NORTE, Trujillo-Peru), and Instituto Regional de Enfermedades Neoplásicas del Sur (IREN-SUR, Arequipa-Peru). Plasma samples were obtained prior to any treatment being administered and underwent analysis using the Bio-Plex Pro™ Human Cytokine 48-Plex Screening Panel kit. Poisson regression models were used to evaluate the association between cytokine levels and complete pathological response (pCR) and clinical response.

**Results:**

Cytokine concentration differences in MIP-1β, IL-9, GRO-α, and TNF-β were observed between BC molecular subtypes. Furthermore, lower levels of circulating FGF BASIC appear necessary to increase the relative risk of achieving pCR (RR = 0.9779; 95% CI 0.9573–0.9989; p=0.0392). Decreased levels of FGF BASIC, PDGF BB, SDF-1, IL-12 P40, and IL-8 were also found to be related to an increased chance of clinical responses to treatments. Multivariable analyses indicated that only FGF-BASIC, PDGF-BB, and IL-12 P40 remained independently associated with clinical response.

**Conclusions:**

Our study identified plasma cytokines linked to BC subtypes and treatment response in a Peruvian cohort. FGF-BASIC consistently demonstrated a significant association with both pCR and clinical response.

## 1 Introduction

Breast cancer (BC) is the leading malignant neoplasm in women across the world. It has been reported more than 2.3 million new cases and approximately 670,000 deaths were recorded in 2022 (1). In Peru, this disease is also the most common cancer among women, with an estimated 7,797 new cases per year and 1,951 deaths from this disease (1).

Apart from hormonal, genetic, and environmental factors, the presence of inflammatory cytokines and proangiogenic factors, such as VEGF, IL-1, IL-6, and IL-8, in the tumor microenvironment stimulates angiogenesis, which in turn promotes tumor growth and metastasis (2, 3). A high level of IL-1β and IL-18 expression is also reported to be linked to poor prognosis due to treatment resistance (4).

Several studies have identified alterations in serum levels of proinflammatory cytokines in BC patients, such as tumor necrosis factor alpha (TNF-α) (5, 6), interleukin 6 (IL-6), and interleukin 1 beta (IL-1β), suggesting that these biomarkers may be involved not only in tumor progression but also in response to treatment (7).

Recent research has suggested that circulating immune mediators could serve as minimally invasive biomarkers to support treatment stratification in BC (8, 9). Blood-based inflammatory markers may help identify patients more likely to respond to treatment (10). However, the clinical utility of cytokine profiling remains uncertain and requires further investigation in diverse populations (11).

Evidence on the cytokine profile in Latin American women, and specifically Peruvian women, is limited. In this context, the present study aims to characterize the inflammatory profile in women with breast cancer in Peru, analyzing plasma levels of inflammatory cytokines and their possible association with clinical variables and therapeutic response. This research aims to enhance the understanding of the immunological mechanisms involved in this pathology and to identify potential prognostic biomarkers and therapeutic targets in this population.

## 2 Materials and methods

### 2.1 Design and study population

Prospective observational cohort study of a longitudinal nature. A total of 103 patients with a confirmed diagnosis of BC were recruited from three cancer centers: Instituto Nacional de Enfermedades Neoplásicas (INEN, Lima-Peru), Instituto Regional de Enfermedades Neoplásicas del Sur (IREN-SUR, Arequipa-Peru), and Instituto Regional de Enfermedades Neoplásicas del Norte (IREN-NORTE, Trujillo-Peru) during November 2023 and December 2024.

### 2.2 Eligibility criteria

Female patients aged 18 years or older with a biopsy- and immunohistochemistry-confirmed diagnosis of and clinical stage II or III disease were included. Those who were pregnant or receiving corticosteroids or prolonged immunosuppressive therapy (more than six months) were excluded. Patients with chronic or inflammatory diseases that could interfere with the immunological analysis and those who had received antibiotic therapy in the 30 days before sample collection were also excluded. Cases in which the biological sample deteriorated during processing or storage were also excluded.

### 2.3 Sample blood collection

Venous blood samples were collected before the start of treatment using a vacuum system in polypropylene tubes containing EDTA K_2_ (6 mL). To obtain plasma, the samples were aliquoted into 2 mL microtubes and centrifuged at 3000 rpm for 10 minutes at room temperature. The supernatant obtained was transferred to new microtubes and initially stored at −20 C until it was transferred to the Institute for Biomedical Research (INICIB). Transport was carried out in containers with dry ice, and once at INICIB, the samples were stored at −80 C until processing and analysis.

### 2.4 Sample processing

Plasma samples were thawed and centrifuged at 10,000 × g for 10 minutes at 4 C. The supernatant obtained was used for cytokine quantification using the Bio-Plex Pro™ Human Cytokine 48-Plex Screening Panel (Bio-Rad) (12), based on multiplex immunoassay technology with magnetic beads (Bio-Plex^®^ Multiplex System) (13). Samples were diluted 1:4, and the standards and controls were prepared according to the manufacturer’s instructions (13).

In each well, 50 µL of beads were added, and then samples, controls, and standards were incubated for 30 minutes at room temperature with constant agitation. After washing, the detection antibodies and streptavidin-phycoerythrin were added. The final complex was resuspended in 125 µL of buffer and shaken for 30 seconds before reading in the Bio-Plex^®^ system, which uses a 532 nm laser to excite the phycoerythrin and measure the mean fluorescence intensity (MFI), proportional to the cytokine concentration (pg/mL). 48 cytokines were evaluated (**Supplementary Table 1**) (13).

### 2.5 Definition of variables

Medical records were reviewed to obtain information on clinical-pathological variables, including age at diagnosis (in years), molecular subtype, clinical stage, and response to treatment.

Molecular subtype was determined by immunohistochemistry (IHC) based on hormone receptor and HER2 status. Luminal cases showed positive expression of estrogen receptors (ER) and/or progesterone receptors (PR) (≥1% of tumor cells with specific nuclear staining). HER2-enriched tumors were defined as HER2-positive, characterized by IHC 3+ or IHC 2+ with amplification confirmed by FISH, and the absence of ER and PR. Triple-negative (TNBC) tumors were characterized by the absence of ER, PR, and HER2 expression (IHC 0–1+ or IHC 2+ without amplification by FISH).

Clinical stage was defined according to the 7th edition of the American Joint Committee on Cancer (AJCC) staging system (14) and categorized as stage II or III at the time of diagnosis.

Response to treatment was evaluated after completion of neoadjuvant chemotherapy. Pathological complete response (pCR) was defined as the absence of residual invasive carcinoma in the breast and lymph nodes (ypT0/is, ypN0). Residual disease was classified using the Residual Cancer Burden (RCB) system. Patients were categorized as responders if they achieved pCR (RCB 0) or partial response (RCB I–III), while patients with stable disease were classified as non-responders.

### 2.6 Statistical analysis

Power analysis was conducted to estimate the minimum detectable effect sizes based on group sizes for both pCR and clinical response outcomes. Calculations assumed 80% power and a two-sided α level of 0.05 (**Supplementary Table 2**). Missing values were primarily attributable to concentrations below the assay detection limit.

Cytokines with more than 20% missing data were excluded from further analyses. For the remaining cytokines, missing values were imputed using a K-nearest neighbors (KNN) methodology, as previously reported in other studies (15, 16), implemented using the *VIM* package in R. Imputation was performed with k = 5 nearest neighbors based on Euclidean distance. Imputation accuracy was evaluated using a masking validation approach, in which artificial missingness was introduced, and the reconstructed values were compared with the observed data. Performance was quantified using the normalized root-mean-square error (NRMSE), defined as the root-mean-square error divided by the standard deviation of the observed values.

Subsequently, base-two logarithm transformation (log_2_[x + 1]) was also applied, consistent with approaches described in cytokine profiling studies (17). Continuous variables were summarized as median values with interquartile ranges (IQR), while categorical variables were summarized as absolute frequencies and percentages. Differences in cytokine concentrations were assessed using boxplots and evaluated with the Mann–Whitney U test for clinical stages and the Kruskal–Wallis test for molecular subtypes. Global expression patterns were explored using hierarchical clustering heatmaps.

Associations between cytokine concentrations and treatment response were evaluated using univariate Poisson regression models with robust variance estimation. Due to the prospective cohort design of the study, relative risks (RR) with corresponding 95% confidence intervals (95% CI) were estimated. Cytokines showing statistical significance in univariate analyses were subsequently included in multivariable models. Statistical significance was defined as p < 0.05. All analyses were performed using R software (version 4.3.1).

### 2.7 Ethics considerations

The study was approved by the Ethics Committees of Ricardo Palma University (No. PI 012-2023), the Instituto Nacional de Enfermedades Neoplásicas (INEN 23-52), the Instituto Regional de Enfermedades Neoplásicas del Norte (No. 021-2023), and the Instituto Regional de Enfermedades Neoplásicas del Sur (No. 062023). The research was conducted in accordance with the ethical principles established in the Declaration of Helsinki. Written informed consent was obtained from all patients whose samples were included in the study.

## 3 Results

### 3.1 General characteristics of patients

**Figure 1:**
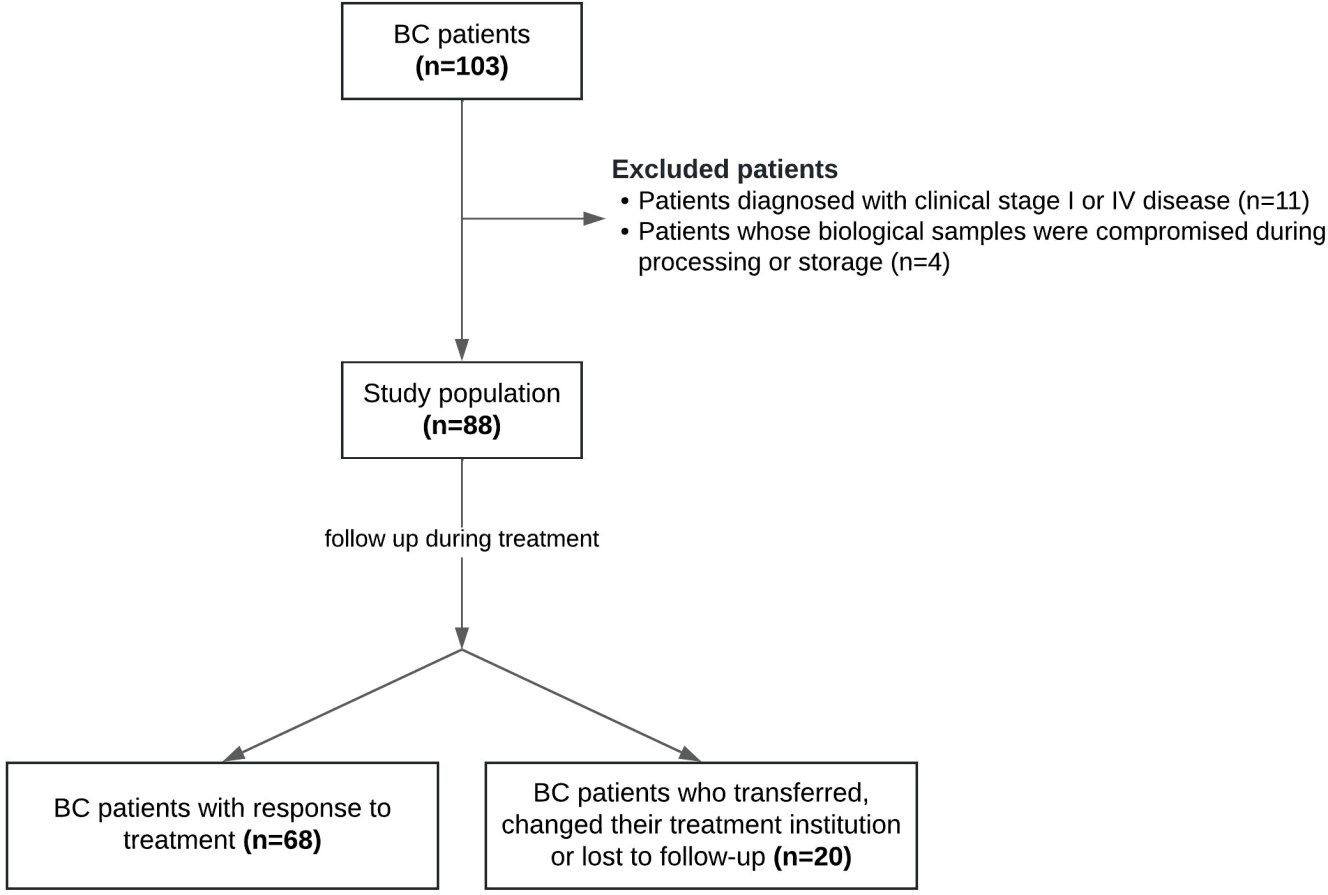
Study flowchart of patient selection.

Of the 103 BC patients, 88 were included (**Figure 1**). Median age was 50 (42.5, 58.5) years. Median body mass index was 26.2 (24.1–28.6) kg/m². 67% were diagnosed in IREN-Norte, followed by INEN (23%) and IREN-Sur (10%). Regarding clinical stage, 55% (n=48) were classified as stage III. The most common subtype was Luminal (58%, n=51), followed by TNBC (24%, n=21) and HER2-enriched (18%, n=16) (**Table 1**).

**Table 1:** Clinicopathologic characteristics of patients.

Characteristic^1^	N = 88^2^
**Age, median (IQR) years**	50 (42.5, 58.5)
Center	
INEN	20 (23%)
IREN-Norte	59 (67%)
IREN-Sur	9 (10%)
**Body mass index, median (IQR) kg/m²**	26.2 (24.1–28.6)
Clinical stage	
II	40 (45%)
III	48 (55%)
Molecular subtype	
HER2-enriched	16 (18%)
Luminal A/B	51 (58%)
TNBC	21 (24%)
HER2 status	
-	68 (77%)
+	20 (23%)
**Treatment regimen**	
AC (Doxorubicin/Adriamycin + Cyclophosphamide)	14 (21%)
AC → T (AC followed by Paclitaxel)	19 (28%)
AC → Carboplatin + Docetaxel	14 (21%)
AC → Carboplatin + Paclitaxel	1 (1.5%)
TC (Docetaxel + Cyclophosphamide)	1 (1.5%)
TCHP (Docetaxel + Carboplatin + Trastuzumab + Pertuzumab)	18 (26%)
TH (Docetaxel + Trastuzumab)	1 (1.5%)
Not available	20
pCR status	
pCR (RCB 0)	10 (15%)
No pCR	58 (65%)
RCB I-III	36
Stable Disease	22
Not available	20
Treatment response	
Responders	46 (68%)
RCB 0	10
RCB I-III	36
Not responders	22 (32%)
Not available	20

^1^ IQR, interquartile range; HER2, human epidermal growth factor receptor 2; TNBC, triple-negative breast cancer; AC, doxorubicin (Adriamycin) plus cyclophosphamide; T, paclitaxel; TC, docetaxel plus cyclophosphamide; TCHP, docetaxel, carboplatin, trastuzumab and pertuzumab; TH, docetaxel plus trastuzumab; pCR, pathological complete response; RCB, residual cancer burden.

^2^ n (%).

Bold values indicate statistical significance (p < 0.05).

Most frequently administered treatment regimen was AC followed by paclitaxel (AC→T), which was received by 28% of patients (n=19). Other commonly used regimens included TCHP (docetaxel, carboplatin, trastuzumab, and pertuzumab) in 26% (n=18), AC alone in 21% (n=14), and AC followed by carboplatin plus docetaxel in 21% (n=14). Less frequently used regimens included AC followed by carboplatin plus paclitaxel, TC (docetaxel plus cyclophosphamide), and TH (docetaxel plus trastuzumab), each administered in 1–2% of patients (**Table 1**).

Treatment response information was available for 68/88 patients. Among these, 46 patients (68%) were classified as responders, including 10 patients who achieved pCR (RCB 0) and 36 patients with partial response (RCB I–III). Around 32% (n=22) were classified as non-responders, corresponding to cases with stable disease (**Table 1**). For the other patients, information was unavailable because some had been transferred or had changed their treatment institution, while others were lost to follow-up at the time of data cutoff. A power analysis showed that only large effects could be detected for pCR, whereas moderate-to-large effects were detectable for clinical response (**Supplementary Table 2**). Accordingly, pCR findings are considered exploratory.

No statistically significant differences were observed in age, treatment center, body mass index, clinical stage, molecular subtype, or HER2 status between patients with and without available treatment response data (**Supplementary Table 3**).

### 3.2 Cytokine levels and profile

After evaluating the missing data, only 42 cytokines were included in the analysis (**Supplementary Table 1**).

**Figure 2:**
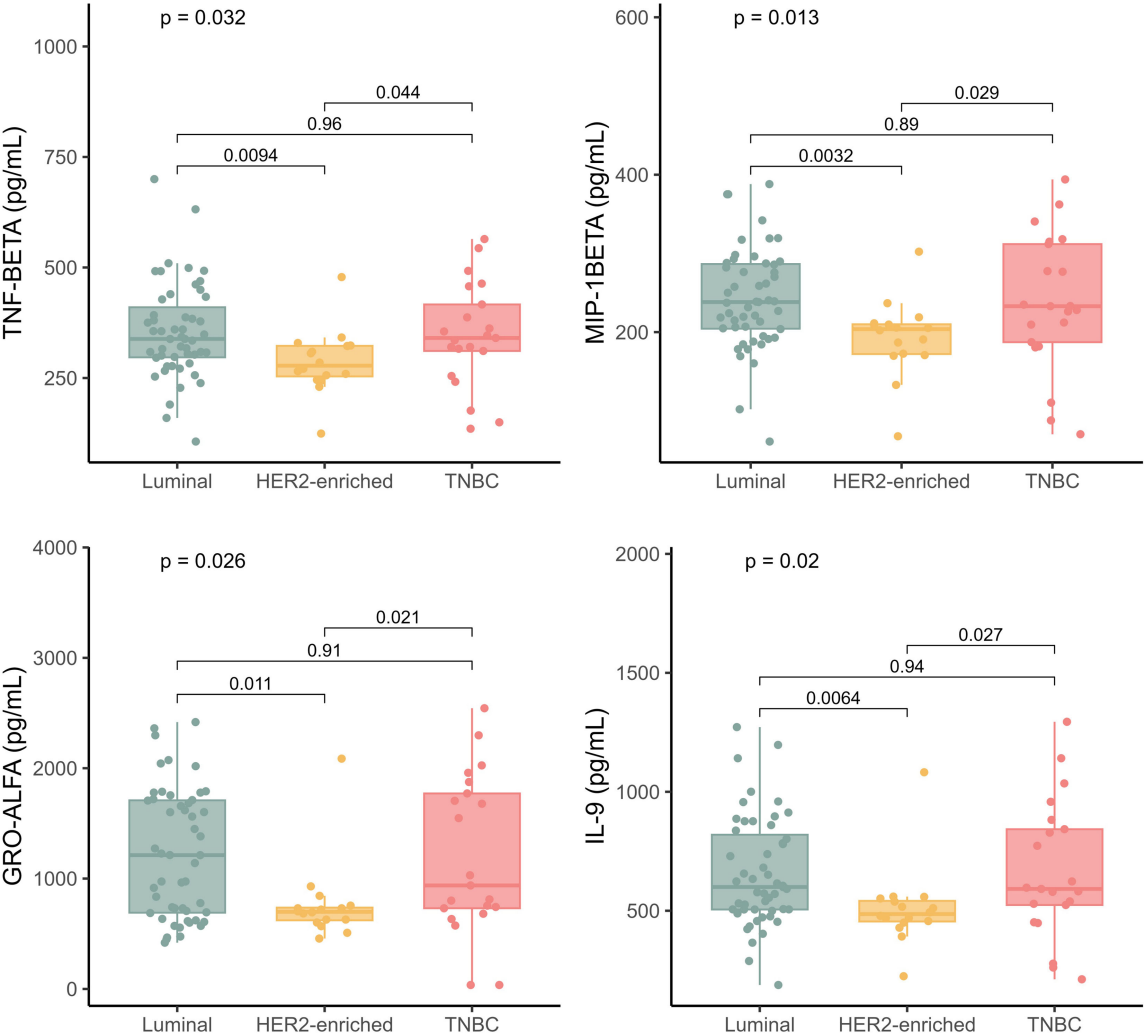
Plasma cytokine profiles according to molecular breast cancer subtype.

Significant differences were observed in plasma levels of the cytokines MIP-1β, IL-9, GRO-α, and TNF-β between molecular subtypes of breast cancer. In general, luminal and TNBC subtypes had higher concentrations. MIP-1β and IL-9 showed higher medians in the Luminal (238.19 and 599.72 pg/mL, respectively) and TNBC (232.93 and 591.74 pg/mL) subtypes, compared to HER2-enriched (203.79 and 486.06 pg/mL; p = 0.0129 and p = 0.0203). Similarly, GRO-α reached higher concentrations in Luminal (1212.16 pg/mL; p = 0.011) and TNBC (937.84 pg/mL; p = 0.021) compared to HER2-enriched (698.61 pg/mL). Finally, TNF-β also remained reduced in the HER2-enriched subtype (277.94 pg/mL) compared to Luminal (338.34 pg/mL; p = 0.0094) and TNBC (340.55 pg/mL; p = 0.044) (**Figure 2**).

**Figure 3:**
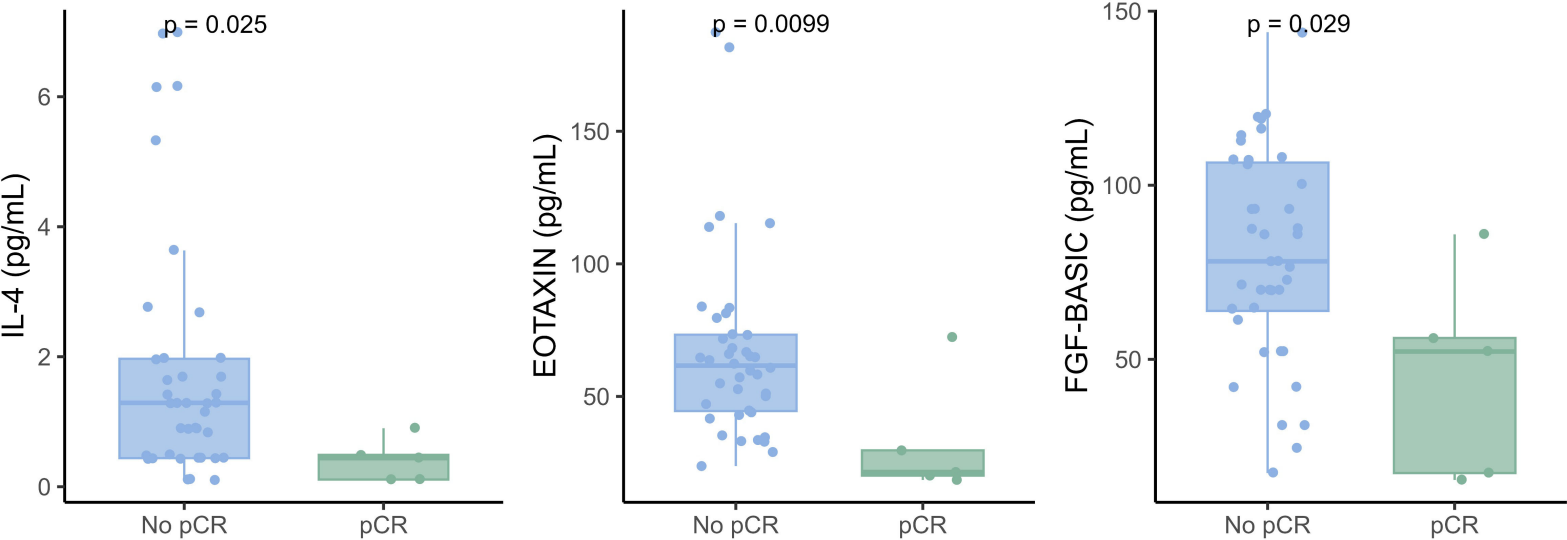
Plasma cytokine profiles according to pathological complete response (pCR) status.

On the other hand, plasma concentrations of Eotaxin, IL-4, and FGF-Basic were significantly lower in patients who achieved pCR compared to those without pCR. Medians for Eotaxin, IL-4, and FGF-Basic were 21.46, 0.44, and 52.23 pg/mL in the pCR group, compared to 61.62, 1.29, and 78.13 pg/mL in the non-pCR group (p=0.010, p=0.025, and p=0.029, respectively) (**Figure 3**).

In addition, concentrations were consistently lower in patients who responded to treatment. Among the cytokines with the greatest differences were BETA-NGF, IL-4, and FGF-Basic, whose levels were significantly reduced in the responder group (0.37, 0.66, and 0.00 pg/mL, respectively) compared to the non-responder group (1.39, 1.70, and 100.51 pg/mL; p < 0.003). Likewise, MIP-1α, G-CSF, IL-8, IFN-γ, GRO-α, and TNF-α showed significant decreases associated with the response. Other cytokines with relevant reductions included IL-6, HGF, MIF, PDGF-BB, and IL-18 (**Table 2**). However, after adjustment for multiple comparisons using the Benjamini–Hochberg false discovery rate (FDR) method, none of these associations remained statistically significant.

**Table 2:** Statistically significant differences in cytokine concentrations between responders and non-responders.

Cytokine	Non-responders (median [IQR] in pg/mL)	Responders (median [IQR] in pg/mL)	P-value^1^
BETA-NGF	1.39 [0.41–1.68]	0.37 [0.04–0.41]	< 0.001
IL-4	1.7 [1.22–2.72]	0.66 [0.44–1.19]	0.002
FGF-BASIC	100.51 [70.81–113.62]	70 [42.23–85.88]	0.003
MIP-1ALFA	1.23 [0.93–1.6]	0.84 [0.31–1.09]	0.007
G-CSF	29.99 [10–46.17]	10 [8.58–16.06]	0.014
IL-8	8.76 [5.31–10]	4.12 [3.01–6.2]	0.018
IFN-GAMMA	29.65 [23.43–43.72]	20.61 [15.68–28.38]	0.020
GRO-ALFA	964.31 [685.82–1249.6]	698.61 [617.94–751.8]	0.022
TNF-ALFA	60.77 [43.27–88.97]	45.95 [35.38–52.96]	0.023
IL-6	1.1 [0.37–2.46]	0.37 [0.37–0.94]	0.027
HGF	269.38 [218.66–330.68]	218.74 [159.66–265.28]	0.027
MIF	681.05 [434.12–1036.39]	429.73 [316.45–611.34]	0.027
PDGF-BB	146.49 [93.12–225.79]	84.03 [48.16–149.7]	0.030
IL-18	45.92 [27.55–66.72]	30.41 [26.25–39]	0.042

^1^ Wilcoxon test.

**Figure 4:**
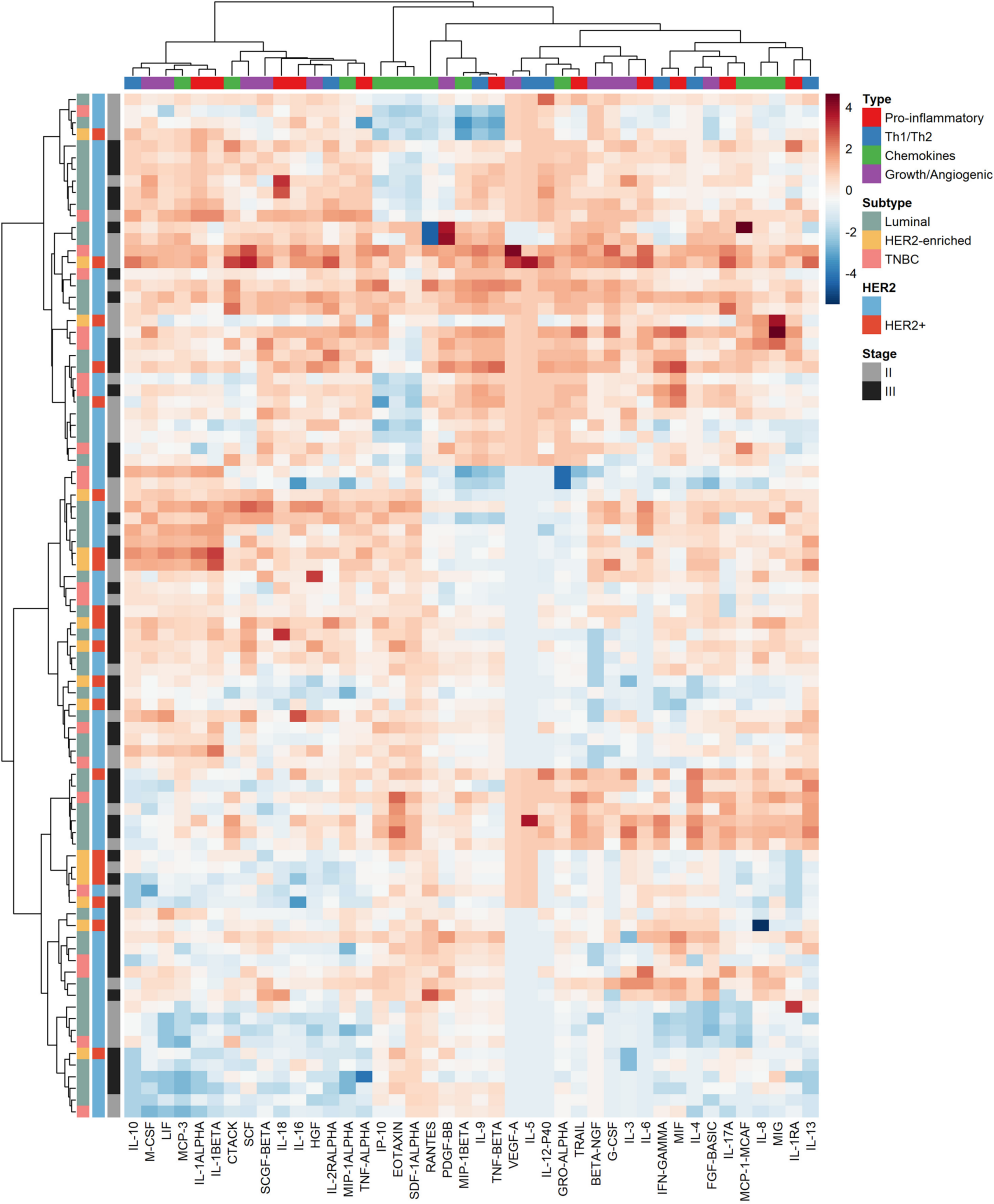
Hierarchical clustering heatmap of plasma cytokine concentrations.

We identified three cytokine clusters based on the heatmap analysis. Cluster 1 comprised IL-3, IL-5, IL-6, IL-8, IL-9, IL-13, IL-17A, VEGF-A, PDGF-BB, IFN-γ, GRO-α, FGF-Basic, MIP-1α, MIP-1β, TNF-β, TRAIL, MCP-1/MCAF, MCP-3, MIF, MIG, and β-NGF. Cluster 2 included IL-10, IL-1α, IL-1β, IL-1RA, IL-16, IL-18, IL-2Rα, IL-12p40, LIF, HGF, G-CSF, M-CSF, SCF, SCGF-β, and TNF-α. Finally, Cluster 3 consisted of Eotaxin, IP-10, CTACK, and SDF-1α (**Figure 4**).

### 3.3 Treatment response

An exploratory analysis stratified by treatment backbone (anthracycline-based and HER2-targeted regimens) showed that, within anthracycline-based therapies, responders had significantly lower baseline levels of IL-4 (median 0.84 pg/mL, IQR 0.44–1.43 vs. 1.70 pg/mL, IQR 1.06–2.77; p = 0.013), FGF-BASIC (66.63 pg/mL, IQR 33.37–85.91 vs. 100.51 pg/mL, IQR 70.00–114.40; p = 0.009), and IL-8 (4.67 pg/mL, IQR 3.20–6.71 vs. 8.82 pg/mL, IQR 6.29–10.67; p = 0.006) than non-responders (**Supplementary Table 4**).

**Figure 5:**
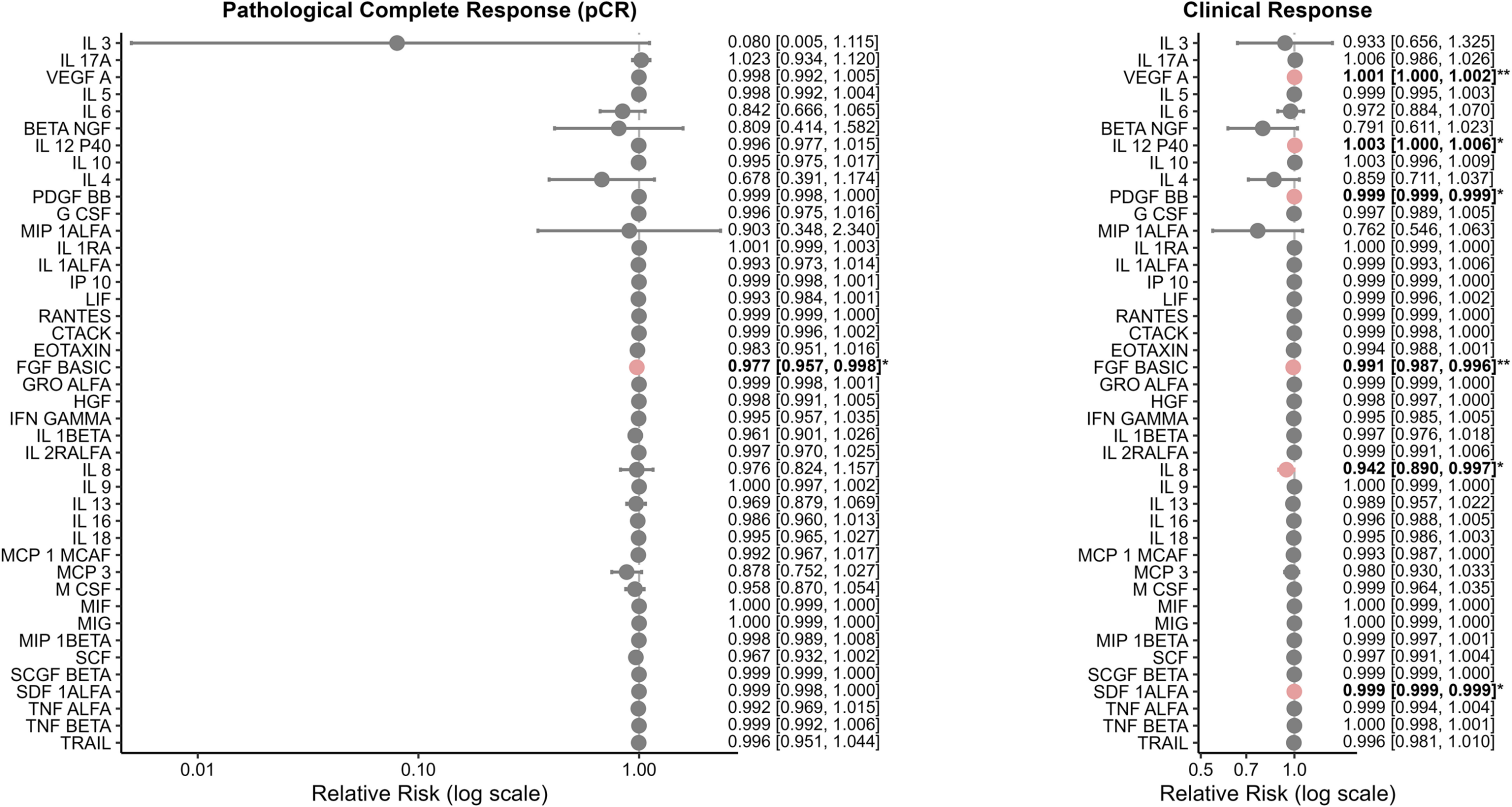
Forest plot of relative risks (RR) and 95% confidence intervals (CI) for the association between plasma cytokine levels and treatment response. For some cytokines, RR values appear close to 1.00 due to rounding; however, these represent small effect sizes derived from continuous variables. Exact values for Multivariable regression are reported in **Table 3**.

Univariate Poisson regression analyses revealed that lower circulating levels of FGF-BASIC were significantly associated with a higher likelihood of achieving pCR (RR = 0.9779; 95% CI 0.9573–0.9989; p=0.0392). Regarding clinical response, lower concentrations of several cytokines were associated with a greater probability of treatment response, including FGF-BASIC (RR = 0.9918; 95% CI 0.9870–0.9968; p=0.001), PDGF-BB (RR = 0.9996; 95% CI 0.9993–0.9999; p=0.011), SDF-1ALFA (RR = 0.9997; 95% CI 0.9994–0.9999; p=0.0224), IL-12 P40 (RR = 1.0032; 95% CI 1.0002–1.0061; p=0.0331), and IL-8 (RR = 0.94; 95% CI 0.89–1.00; p = 0.041) (**Figure 5**). In the multivariable model for clinical response, only FGF-BASIC (RR = 0.9929; 95% CI 0.9869–0.9990; p=0.022), PDGF-BB (RR = 0.9997; 95% CI 0.9994–0.9999; p=0.013), and IL-12p40 (RR = 1.0083; 95% CI 1.0030–1.0136; p=0.002) remained significantly associated with response (**Table 3**). Importantly, these associations remained consistent after additional adjustment for molecular subtype (**Supplementary Table 5**).

**Table 3:** Multivariable poisson regression for clinical response.

Cytokine	RR^1^	95% CI^1^	P-value
FGF BASIC	0.9929	[0.9869, 0.9990]	**0.022**
VEGF A	1.0008	[0.9988, 1.0028]	0.419
PDGF BB	0.9997	[0.9994, 0.9999]	**0.013**
SDF 1ALFA	0.9999	[0.9997, 1.0002]	0.957
IL 12 P40	1.0083	[1.0030, 1.0136]	**0.002**
IL 8	0.9505	[0.8844, 1.0216]	0.169

^1^ Relative Risk (RR), 95% Confidence Interval (95% CI).

Bold values indicate statistical significance (p < 0.05).

According to analysis stratified by molecular subtype, associations between circulating cytokines and treatment response were heterogeneous. In luminal tumors, several cytokines were significantly associated with clinical response, including lower levels of FGF-BASIC (RR = 0.990, 95% CI 0.983–0.997; p=0.006), IL-6 (RR = 0.761, 95% CI 0.609–0.951; p=0.016), IL-8 (RR = 0.871, 95% CI 0.764–0.992; p=0.037), SDF-1ALFA (RR = 0.9996, 95% CI 0.9992–0.9999; p=0.017), PDGF-BB (RR = 0.9996, 95% CI 0.9993–0.99997; p=0.031), MIP-1ALFA (RR = 0.611, 95% CI 0.378–0.986; p=0.044), and BETA-NGF (RR = 0.656, 95% CI 0.441–0.977; p=0.038) (**Supplementary Table 6**). In HER2-enriched tumors, no cytokines were significantly associated with clinical response; however, in the pCR analysis, several markers were significantly associated with higher likelihood of pCR, including FGF-BASIC (RR = 0.950, 95% CI 0.928–0.973; p<0.001), EOTAXIN (RR = 0.901, 95% CI 0.855–0.949; p<0.001), IL-4 (RR = 0.252, 95% CI 0.078–0.810; p=0.021), SCF (RR = 0.928, 95% CI 0.873–0.987; p=0.017), and IL-18 (RR = 0.918, 95% CI 0.850–0.993; p=0.032) (**Supplementary Table 7**). In TNBC, although no cytokines were significantly associated with clinical response, multiple cytokines were strongly associated with pCR, including FGF-BASIC (RR = 0.242, 95% CI 0.215–0.272; p<0.001), IL-2RALFA (RR = 0.032, 95% CI 0.024–0.043; p<0.001), IFN-GAMMA (RR = 0.0039, 95% CI 0.0023–0.0065; p<0.001), HGF (RR = 0.245, 95% CI 0.215–0.280; p<0.001), and IL-16 (RR = 0.342, 95% CI 0.322–0.364; p<0.001) (**Supplementary Table 7**).

Among these different analysis approaches, FGF-BASIC showed a consistent association with both pCR and clinical response.

## 4 Discussion

In this study, we identified cytokine levels associated both with molecular subtypes and with treatment response. Among these, FGF-Basic emerged as a cytokine showing a consistent association with both pCR and clinical response.

Our study population was predominantly characterized by luminal tumors, which is consistent with previous reports indicating that luminal A/B subtypes are the most prevalent (42–59%), followed by triple-negative breast cancer (TNBC, 15–24%) and HER2-enriched tumors (15–18%). This trend has been consistently observed across diverse populations and multicenter studies (18–20).

Consequently, the lower pCR rate observed may be partly attributable to the predominance of the luminal-type tumors. pCR rates are variable depending on the molecular subtype of the tumor. TNBC and HER2-enriched tumors tend to have higher pCR rates (28%-45%), while luminal subtype tumors demonstrate much lower rates (4%-12%) (20–22), mainly due to increased sensitivity to chemotherapy among TNBC and HER2-enriched cells.

Furthermore, luminal and TNBC subtypes exhibited higher plasma levels of specific cytokines, including MIP-1β, IL-9, GRO-α, and TNF-β, compared with the HER2-enriched subtype. Our findings suggest that these two types of tumors may have more active immunological and inflammatory profiles than HER2-enriched tumors. However, most existing studies indicate that luminal subtype tumors (ER+/HER2-) are immunologically cold tumors, meaning they have very few immune cells within their stromal compartment, characterized by low immune cell infiltration and a predominance of M2 macrophages and regulatory T cells (Tregs) that promote immunosuppression (23). Conversely, TNBC tumors tend to produce higher amounts of TILs, resulting in increased pro-inflammatory cytokines and greater immunogenicity. MIP-1β is key to recruitment of immune cells, especially lymphocytes and macrophages, and is linked with increased immune infiltration and aggressiveness in ER-negative and high-grade tumors (24, 25). In particular, MIP-1β plays a central role in the recruitment of immune cells such as lymphocytes and macrophages and has been associated with increased immune infiltration and tumor aggressiveness, especially in ER-negative and high-grade tumors (26, 27).

Even though many previous reports have demonstrated an association between elevated TGF-β levels and patients with luminal and HER2-enriched tumors (28), the role of TGF-β in TNBC is still unclear. Some studies reported that TGF-β is associated with increased invasion/metastasis in TNBC (29). However, other studies have shown that TGF-β may have a tumor-suppressive effect during early stages of TNBC due to the induction of apoptosis or senescence (30). Discrepancies between our findings and prior studies may be partly explained by differences in the type of biological samples analyzed.

Additionally, we report that lower levels of IL-4 and basic FGF found in plasma are associated with a greater response to treatment. These findings correlate with previous evidence that suggests Th2/IL-4 signaling contributes to immunosuppressive microenvironments in tumors, leading to increased tumor survival and metastatic potential, therefore resulting in a poorer prognosis (31). β-NGF has also been reported to demonstrate a similar pattern (32).

In this context, our study demonstrated that lower concentrations of FGF-Basic were significantly associated with improved treatment response. Biological role of FGF-Basic is largely defined by its pro-angiogenic and mitogenic properties, which are implicated in tumor progression. Both clinical and preclinical studies have shown that FGF-Basic, also known as FGF2, can promote an immunosuppressive tumor microenvironment. Im et al. reported that the absence of FGF2 in murine models was associated with enhanced activation of inflammatory macrophages and reduced tumor growth (33). Additionally, a clinical study demonstrated that low tumoral expression of FGF2 correlated with improved response to neoadjuvant chemotherapy consisting of docetaxel, doxorubicin, and cyclophosphamide (34).

However, in a phase II clinical trial evaluating the addition of antiangiogenic therapy (bevacizumab) to neoadjuvant chemotherapy, elevated baseline serum levels of FGF2 were significantly associated with the achievement of pCR (35). It was hypothesized that tumors with high FGF2 levels possess an intrinsically fragile and disorganized vasculature dependent on multiple concurrent signaling pathways. In this context, VEGF inhibition with bevacizumab may induce a more pronounced vascular collapse, resulting in higher pCR rates. Accordingly, low FGF2 levels appear to be associated with improved response in the setting of standard cytotoxic therapy, whereas elevated FGF2 levels may become predictive only in the context of antiangiogenic treatment. Therefore, the prognostic value of FGF2 is not fixed but rather depends on the therapeutic strategy employed.From a clinical perspective, identifying circulating cytokines associated with treatment response may contribute to the development of minimally invasive biomarkers to support patient stratification before therapy. In particular, the consistent association between lower circulating FGF-Basic levels and improved response suggests that angiogenic and immune-related pathways may influence the effectiveness of neoadjuvant chemotherapy (34). Our findings support the growing interest in circulating immune mediators as indicators of tumor–host interactions that could complement existing clinicopathological factors when evaluating treatment response.

## 5 Limitations

Although our study had a prospective design that enabled longitudinal patient follow-up, some limitations should be acknowledged. First, even though patients were recruited from three different Peruvian cancer centers, the analyses were conducted using a pooled cohort because of the relatively small sample size. Consequently, the study was not able to provide external validation of the associations between plasma cytokine levels and therapeutic response across independent cohorts, and the findings should therefore be interpreted as exploratory. In addition, the limited sample size may have reduced the statistical power to detect certain associations. Furthermore, after applying false discovery rate (FDR) correction for descriptive tests, none of the observed associations remained statistically significant, reinforcing the exploratory nature of these results.

Treatment response data were unavailable for a proportion of patients. Baseline clinical characteristics did not differ significantly between patients with and without available response information, suggesting that the analytical sample was broadly comparable to the overall cohort. Nevertheless, the potential impact of missing outcome data cannot be excluded, and some degree of selection bias may remain. In addition, the number of pCR events in our cohort was limited, which restricted the ability to perform robust multivariable analyses for this outcome. Therefore, the observed associations between baseline cytokine levels and pCR, particularly for Eotaxin, IL-4, and FGF-Basic, should be interpreted as exploratory.

Additionally, cytokine concentrations were measured at a single pre-treatment time point; therefore, we were not able to assess dynamic changes or chemotherapy-induced modulation over time. Thus, while the study focused on circulating cytokines, information regarding their tissue-level correlates or the composition of the tumor microenvironment was not available, which may have constrained the biological interpretation of the findings.

Also, subtype-stratified analyses should be interpreted with caution due to the limited sample size and the small number of events within each subgroup, particularly for pCR in TNBC and HER2-enriched tumors, which may have led to unstable estimates and inflated effect sizes. Finally, the observed subtype-specific associations should be considered exploratory and hypothesis-generating rather than definitive.

Despite these limitations, this study provides novel evidence supporting the potential value of plasma cytokine profiling as a biomarker of therapeutic response in breast cancer. Future prospective studies with larger sample sizes and longer follow-up periods are warranted to evaluate the association between cytokine levels and clinically relevant outcomes, including overall survival and progression-free survival.

## 6 Conclusion

Our study identified a plasma cytokine profile associated with both molecular subtypes of BC and the likelihood of therapeutic response in the context of standard chemotherapy, consistently highlighting FGF-Basic as a relevant biomarker. Future studies should validate these findings in larger cohorts and investigate whether baseline cytokine levels and post-treatment changes hold prognostic value for clinical outcomes, including overall survival and progression-free survival.

## Data Availability

The raw data supporting the conclusions of this article will be made available by the authors, without undue reservation.
