# Supplementary material for: Plasma cytokine profiles in breast cancer patients and their association with therapeutic response in Peru: a prospective cohort study
Source: Front Immunol. 2026 Jun 9;17:1771790. doi: 10.3389/fimmu.2026.1771790 (PMC13287038; doi:10.3389/fimmu.2026.1771790)
Supplement: Supplementary file 1 [file Table1.docx]

**Table S1:** Bio-Plex Pro™ Human Cytokine 48-Plex Screening Panel

| **Abbreviation** | **Cytokine full name** |
| --- | --- |
| FGF | Fibroblast Growth Factor |
| CTACK | Cutaneous T Cell-Attracting Chemokine (CCL27) |
| Eotaxin | Eotaxin (CCL11) |
| G-CSF | Granulocyte Colony-Stimulating Factor |
| GM-CSF | Granulocyte-Macrophage Colony-Stimulating Factor |
| GRO-α | Growth-Regulated Oncogene Alpha (CXCL1) |
| HGF | Hepatocyte Growth Factor |
| IFN-α2 | Interferon Alpha-2 |
| IFN-γ | Interferon Gamma |
| IL-1α | Interleukin 1 Alpha |
| IL-1β | Interleukin 1 Beta |
| IL-1ra | Interleukin 1 Receptor Antagonist |
| IL-2 | Interleukin 2 |
| IL-2Rα | Interleukin 2 Receptor Alpha |
| IL-3 | Interleukin 3 |
| IL-4 | Interleukin 4 |
| IL-5 | Interleukin 5 |
| IL-6 | Interleukin 6 |
| IL-7 | Interleukin 7 |
| IL-8 | Interleukin 8 (CXCL8) |
| IL-9 | Interleukin 9 |
| IL-10 | Interleukin 10 |
| IL-12 (p40) | Interleukin 12 Subunit p40 |
| IL-12 (p70) | Interleukin 12 Heterodimer (p35/p40) |
| IL-13 | Interleukin 13 |
| IL-15 | Interleukin 15 |
| IL-16 | Interleukin 16 |
| IL-17A | Interleukin 17A |
| IL-18 | Interleukin 18 |
| IP-10 | Interferon Gamma-Induced Protein 10 (CXCL10) |
| LIF | Leukemia Inhibitory Factor |
| MCP-1 (MCAF) | Monocyte Chemoattractant Protein 1 (Monocyte Chemotactic and Activating Factor, CCL2) |
| MCP-3 | Monocyte Chemoattractant Protein 3 (CCL7) |
| M-CSF | Macrophage Colony-Stimulating Factor |
| MIF | Macrophage Migration Inhibitory Factor |
| MIG | Monokine Induced by Gamma Interferon (CXCL9) |
| MIP-1α | Macrophage Inflammatory Protein 1 Alpha (CCL3) |
| MIP-1β | Macrophage Inflammatory Protein 1 Beta (CCL4) |
| β-NGF | Beta Nerve Growth Factor |
| PDGF-BB | Platelet-Derived Growth Factor BB |
| RANTES | Regulated upon Activation, Normal T Cell Expressed and Secreted (CCL5) |
| SCF | Stem Cell Factor |
| SCGF-β | Stem Cell Growth Factor Beta |
| SDF-1α | Stromal Cell-Derived Factor 1 Alpha (CXCL12) |
| TNF-α | Tumor Necrosis Factor Alpha |
| TNF-β | Tumor Necrosis Factor Beta (Lymphotoxin Alpha) |
| TRAIL | TNF-Related Apoptosis-Inducing Ligand |
| VEGF-A | Vascular Endothelial Growth Factor A |
| **Cytokines highlighted in red were not included in the analysis due to exclusion criteria applied during data preprocessing.** | |

**Table S2:** Power analysis for primary and secondary outcomes

| **Outcome** | **Prevalence** | **Detectable effect size (Cohen’s d)** |
| --- | --- | --- |
| pCR | 14.7% | 0.97 |
| Clinical response | 67.6% | 0.74 |

**Figure S1:** Imputation performance across cytokines assessed by masking validation (NRMSE)

**
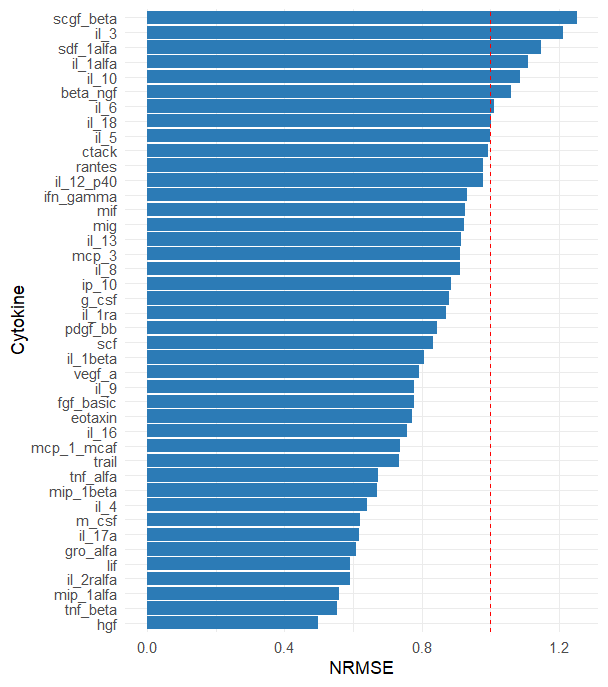
**

**Table S3:** Baseline characteristics of patients with and without treatment response data.

| **Characteristic** | **Overall**  **N = 88**^1^ | **Available treatment response data**  **N = 68**^1^ | **Not available treatment response data**  **N = 20**^1^ | **p-value**^2^ |
| --- | --- | --- | --- | --- |
| **Age, median (IQR) years** | 50 (42.5, 58.5) | 49.5 (43.0, 59.0) | 50.0 (42.0, 56.5) | 0.7 |
| **Center** |  |  |  | 0.11 |
| INEN | 20 (23%) | 13 (19%) | 7 (35%) |  |
| IREN-Norte | 59 (67%) | 46 (68%) | 13 (65%) |  |
| IREN-Sur | 9 (10%) | 9 (13%) | 0 (0%) |  |
| **Body mass index, median (IQR) kg/m²** | 26.2 (24.1–28.6) | 26.2 (24.0, 28.6) | 26.3 (24.2, 28.6) | 0.8 |
| **Clinical stage** |  |  |  | 0.14 |
| II | 40 (45%) | 28 (41%) | 12 (60%) |  |
| III | 48 (55%) | 40 (59%) | 8 (40%) |  |
| **Molecular subtype** |  |  |  | 0.6 |
| HER2-enriched | 16 (18%) | 14 (21%) | 2 (10%) |  |
| Luminal A/B | 51 (58%) | 38 (56%) | 13 (65%) |  |
| TNBC | 21 (24%) | 16 (24%) | 5 (25%) |  |
| **HER2 status** |  |  |  | 0.14 |
| - | 68 (77%) | 50 (74%) | 18 (90%) |  |
| + | 20 (23%) | 18 (26%) | 2 (10%) |  |
| ^1^Median (Q1, Q3); n (%)  ^2^Wilcoxon rank sum test; Fisher’s exact test; Pearson’s Chi-squared test | | | | |

**Table S4:** Comparison of baseline cytokine levels between responders and non-responders stratified by treatment backbone (anthracycline-based and HER2-targeted regimens).

| **Cytokines** | **Anthracycline-based** | | | **HER2-targeted** | | |
| --- | --- | --- | --- | --- | --- | --- |
|  | **Non-responder**  **N = 17**^1^ | **Responder**  **N = 31**^1^ | **p-value**^2^ | **Non-responder**  **N = 4**^1^ | **Responder**  **N = 15**^1^ | **p-value**^2^ |
| IL-3 | 0.34 (0.14, 0.56) | 0.14 (0.10, 0.45) | 0.3 | 0.17 (0.08, 0.28) | 0.27 (0.11, 0.34) | 0.4 |
| IL-17A | 5.60 (4.32, 9.01) | 5.60 (2.20, 8.87) | 0.7 | 3.49 (2.51, 5.89) | 5.60 (2.78, 9.64) | 0.4 |
| VEGF-A | 11.10 (11.10, 39.65) | 11.10 (11.10, 39.65) | 0.7 | 11.10 (11.10, 25.38) | 11.10 (11.10, 39.65) | 0.5 |
| IL-5 | 10.72 (10.72, 42.89) | 10.72 (10.72, 42.89) | 0.8 | 10.72 (10.72, 26.80) | 10.72 (10.72, 42.89) | 0.5 |
| IL-6 | 1.47 (0.37, 3.76) | 0.47 (0.37, 1.71) | 0.2 | 0.74 (0.37, 1.51) | 1.13 (0.37, 1.91) | 0.6 |
| BETA-NGF | 0.89 (0.41, 1.94) | 0.52 (0.19, 1.15) | 0.081 | 0.96 (0.37, 1.53) | 0.41 (0.04, 1.58) | 0.8 |
| IL-12-P40 | 11.75 (9.00, 46.91) | 26.63 (9.00, 46.06) | 0.5 | 10.38 (8.72, 15.95) | 11.75 (9.00, 46.06) | 0.4 |
| IL-10 | 7.16 (1.17, 10.88) | 8.81 (5.78, 13.38) | 0.4 | 22.65 (10.96, 31.14) | 11.14 (1.88, 24.09) | 0.4 |
| IL-4 | 1.70 (1.06, 2.77) | 0.84 (0.44, 1.43) | **0.013** | 1.29 (0.89, 1.99) | 0.90 (0.49, 2.15) | 0.5 |
| PDGF-BB | 152.11 (135.84, 383.19) | 107.26 (43.99, 276.28) | 0.064 | 81.12 (48.91, 124.36) | 84.21 (72.21, 249.12) | 0.5 |
| G-CSF | 20.31 (10.00, 43.28) | 17.39 (10.00, 27.15) | 0.6 | 32.52 (18.46, 62.39) | 26.55 (10.00, 35.92) | 0.5 |
| MIP-1ALFA | 1.23 (1.09, 1.42) | 1.02 (0.57, 1.23) | 0.1 | 1.50 (0.95, 1.79) | 1.01 (0.57, 1.29) | 0.3 |
| IL-1RA | 201.14 (148.01, 272.63) | 174.98 (118.37, 223.68) | 0.5 | 223.10 (128.12, 263.60) | 200.05 (118.37, 233.88) | 0.7 |
| IL-1ALFA | 28.20 (17.02, 38.22) | 28.27 (18.26, 42.38) | 0.6 | 57.79 (32.70, 79.83) | 31.42 (22.30, 69.49) | 0.5 |
| IP-10 | 424.44 (265.18, 604.81) | 337.83 (233.99, 614.48) | 0.7 | 494.67 (243.67, 824.98) | 316.30 (204.93, 464.53) | 0.5 |
| LIF | 62.63 (25.34, 96.59) | 48.67 (33.47, 75.75) | 0.7 | 128.09 (62.83, 164.63) | 79.59 (37.13, 134.40) | 0.6 |
| RANTES | 9,885.11 (7,313.26, 16,669.47) | 7,266.74 (4,057.13, 17,186.54) | 0.4 | 7,262.00 (3,347.88, 11,617.38) | 9,133.98 (3,009.44, 17,168.91) | 0.7 |
| CTACK | 369.25 (222.47, 604.22) | 266.77 (193.75, 383.58) | 0.2 | 276.83 (223.59, 341.22) | 306.76 (202.49, 398.40) | 0.9 |
| EOTAXIN | 62.38 (44.65, 79.66) | 39.89 (28.37, 67.11) | 0.077 | 61.49 (54.20, 64.92) | 56.08 (29.66, 73.46) | 0.6 |
| FGF-BASIC | 100.51 (70.00, 114.40) | 66.63 (33.37, 85.91) | **0.009** | 89.61 (75.30, 100.69) | 70.00 (52.23, 99.00) | 0.6 |
| GRO-ALFA | 1,213.30 (915.83, 1,683.36) | 835.31 (681.33, 1,778.92) | 0.5 | 642.91 (533.81, 707.47) | 719.38 (614.47, 1,657.56) | 0.2 |
| HGF | 283.63 (223.59, 343.02) | 233.78 (193.60, 295.03) | 0.13 | 281.47 (218.62, 313.36) | 278.66 (163.26, 382.39) | >0.9 |
| IFN-GAMMA | 35.44 (23.90, 46.77) | 27.67 (15.45, 34.30) | 0.091 | 29.37 (23.90, 32.91) | 24.65 (18.26, 51.59) | 0.9 |
| IL-1BETA | 6.67 (5.43, 12.53) | 9.34 (5.76, 12.42) | 0.6 | 19.50 (12.84, 33.25) | 8.86 (5.11, 14.16) | 0.08 |
| IL-2RALFA | 37.20 (28.91, 65.47) | 39.26 (30.29, 51.95) | >0.9 | 39.35 (32.30, 69.95) | 41.77 (30.98, 62.82) | >0.9 |
| IL-8 | 8.82 (6.29, 10.67) | 4.67 (3.20, 6.71) | **0.006** | 5.50 (4.61, 9.89) | 4.48 (2.65, 7.40) | 0.4 |
| IL-9 | 605.07 (506.83, 801.88) | 577.81 (472.13, 828.27) | 0.7 | 451.87 (431.40, 514.09) | 510.90 (470.08, 681.47) | 0.2 |
| IL-13 | 5.30 (2.29, 7.07) | 4.18 (2.39, 7.31) | >0.9 | 11.07 (5.62, 17.65) | 5.76 (4.18, 9.98) | 0.3 |
| IL-16 | 43.76 (27.69, 60.02) | 37.38 (29.86, 43.76) | 0.2 | 36.80 (32.05, 47.76) | 42.51 (29.66, 62.65) | 0.7 |
| IL-18 | 40.99 (27.43, 66.48) | 39.28 (29.53, 60.42) | 0.7 | 54.28 (40.43, 70.19) | 34.48 (24.34, 60.58) | 0.12 |
| MCP-1-MCAF | 11.50 (9.06, 19.20) | 11.76 (7.19, 18.54) | 0.6 | 10.79 (8.58, 13.27) | 12.01 (6.06, 17.09) | 0.8 |
| MCP-3 | 4.18 (3.09, 5.62) | 3.77 (2.01, 5.62) | 0.5 | 9.02 (4.23, 11.69) | 3.77 (1.67, 6.92) | 0.2 |
| M-CSF | 7.48 (3.53, 11.30) | 7.48 (5.20, 9.98) | 0.9 | 14.75 (9.88, 16.43) | 8.71 (5.30, 12.37) | 0.3 |
| MIF | 681.05 (472.70, 1,348.26) | 559.49 (381.13, 1,093.87) | 0.3 | 602.87 (390.29, 944.58) | 578.71 (433.18, 1,167.32) | 0.9 |
| MIG | 445.90 (376.74, 638.13) | 405.41 (255.21, 622.58) | 0.4 | 382.97 (239.75, 848.23) | 346.42 (219.38, 679.64) | >0.9 |
| MIP-1BETA | 239.15 (214.88, 311.85) | 228.10 (206.15, 277.50) | 0.3 | 186.09 (169.65, 210.56) | 209.12 (172.58, 296.21) | 0.3 |
| SCF | 62.04 (58.69, 83.76) | 64.63 (45.31, 80.08) | 0.6 | 93.85 (74.34, 103.85) | 57.57 (50.17, 75.35) | 0.1 |
| SCGF-BETA | 80,540.05 (56,594.32, 108,658.50) | 65,988.32 (51,447.10, 88,195.28) | 0.3 | 70,663.07 (50,405.54, 85,624.26) | 72,687.96 (52,418.10, 91,418.18) | 0.8 |
| SDF-1ALFA | 1,703.75 (1,327.36, 2,219.29) | 1,534.60 (624.45, 1,819.01) | 0.2 | 1,662.47 (1,443.50, 1,905.12) | 1,458.88 (1,037.02, 1,970.99) | 0.5 |
| TNF-ALFA | 59.20 (46.85, 92.29) | 51.57 (38.30, 85.68) | 0.4 | 69.71 (49.99, 88.85) | 75.11 (45.27, 85.63) | >0.9 |
| TNF-BETA | 342.81 (308.92, 433.60) | 338.31 (266.30, 392.25) | 0.5 | 273.65 (258.36, 309.02) | 321.36 (259.35, 348.49) | 0.5 |
| TRAIL | 37.56 (26.30, 43.82) | 27.18 (23.57, 41.95) | 0.3 | 24.08 (22.70, 28.44) | 26.17 (24.16, 39.35) | 0.2 |
| ^1^Median (Q1, Q3)  ^2^Wilcoxon rank sum test | | | | | |  |

**Table S5:** Multivariable Poisson Regression for Clinical Response adjusted by molecular subtype

| **Cytokine** | **Adjusted RR**^1^ | **95% CI**^1^ | **p-value** |
| --- | --- | --- | --- |
| FGF BASIC | 0.9931 | [0.9869, 0.9994] | **0.0332** |
| VEGF A | 0.9999 | [0.9999, 1.0022] | 0.9994 |
| PDGF BB | 0.9997 | [0.9995, 0.9999] | **0.0189** |
| SDF 1ALFA | 1.0000 | [0.9997, 1.0002] | 0.9087 |
| IL 12 P40 | 1.0094 | [1.0040, 1.0147] | **0.0005** |
| IL 8 | 0.9485 | [0.8872, 1.014] | 0.1219 |
| ^1^Adjusted Relative Risk (RR), 95% Confidence Interval (95% CI) by molecular type (Reference: Luminal, HER2-enriched RR = 1.2929 (95% CI [0.9108, 1.8354], p = 0.1507) and TNBC RR = 1.3665 (95% CI [0.9841, 1.8976], p = 0.0623). | | | |

**Table S6:** Univariable Poisson regression for clinical response stratified by molecular subtype

| **Cytokine** | **HER2-enriched**^1^  n=14 (11 responders/3 non-responders) | **Luminal**^1^  n=38 (22 responders/16 non-responders) | **TNBC**^1^  n=16 (13 responders/3 non-responders) |
| --- | --- | --- | --- |
| IL-18 | 0.98326 [0.96349, 1.00344]  p=0.10331 | 0.99673 [0.98460, 1.00901]  p=0.60003 | 1.00255 [0.99068, 1.01457]  p=0.67494 |
| IL-12-P40 | 1.00213 [0.99954, 1.00472]  p=0.10689 | 1.00600 [1.00074, 1.01128]  **p=0.02520** | 0.99957 [0.99253, 1.00667]  p=0.90595 |
| IL-5 | 1.00074 [0.99978, 1.00171]  p=0.13133 | 0.99788 [0.99152, 1.00427]  p=0.51455 | 0.98549 [0.96919, 1.00208]  p=0.08612 |
| PDGF-BB | 1.00044 [0.99986, 1.00103]  p=0.14013 | 0.99962 [0.99928, 0.99997]  **p=0.03094** | 0.99957 [0.99844, 1.00069]  p=0.45102 |
| GRO-ALFA | 1.00018 [0.99994, 1.00042]  p=0.14709 | 1.00017 [0.99974, 1.00060]  p=0.43189 | 0.99982 [0.99951, 1.00014]  p=0.27400 |
| IL-17A | 1.00952 [0.99595, 1.02328]  p=0.17014 | 1.01182 [0.97288, 1.05232]  p=0.55734 | 0.99948 [0.96436, 1.03588]  p=0.97716 |
| IL-6 | 1.02775 [0.98511, 1.07225]  p=0.20549 | 0.76063 [0.60861, 0.95061]  **p=0.01616** | 1.00362 [0.95306, 1.05687]  p=0.89087 |
| IL-3 | 1.10030 [0.94341, 1.28328]  p=0.22329 | 0.97017 [0.59718, 1.57612]  p=0.90263 | 0.80992 [0.14224, 4.61176]  p=0.81223 |
| FGF-BASIC | 0.99380 [0.98372, 1.00399]  p=0.23226 | 0.99023 [0.98328, 0.99724]  **p=0.00634** | 0.99520 [0.98759, 1.00287]  p=0.21935 |
| VEGF-A | 1.00145 [0.99903, 1.00388]  p=0.24035 | 1.01093 [0.99929, 1.02271]  p=0.06590 | 1.00021 [0.99933, 1.00109]  p=0.63728 |
| CTACK | 1.00020 [0.99984, 1.00055]  p=0.28391 | 0.99971 [0.99838, 1.00104]  p=0.66484 | 0.99853 [0.99570, 1.00137]  p=0.30974 |
| MIP-1ALFA | 0.75824 [0.45603, 1.26074]  p=0.28605 | 0.61088 [0.37846, 0.98604]  **p=0.04364** | 1.00637 [0.74471, 1.35996]  p=0.96705 |
| MCP-1-MCAF | 1.00739 [0.99370, 1.02127]  p=0.29172 | 0.99099 [0.98052, 1.00157]  p=0.09469 | 0.99518 [0.97505, 1.01572]  p=0.64305 |
| MIG | 1.00004 [0.99996, 1.00011]  p=0.31438 | 0.99911 [0.99781, 1.00042]  p=0.18498 | 1.00000 [0.99993, 1.00006]  p=0.89816 |
| SCGF-BETA | 1.00000 [1.00000, 1.00001]  p=0.37094 | 0.99999 [0.99998, 1.00000]  p=0.25884 | 1.00000 [0.99999, 1.00001]  p=0.76376 |
| M-CSF | 0.97447 [0.91897, 1.03332]  p=0.38733 | 0.98246 [0.91844, 1.05095]  p=0.60682 | 1.02977 [0.98788, 1.07343]  p=0.16621 |
| G-CSF | 0.99256 [0.97589, 1.00951]  p=0.38735 | 0.99302 [0.97579, 1.01056]  p=0.43295 | 1.00126 [0.99812, 1.00442]  p=0.43150 |
| IL-1BETA | 0.98684 [0.95445, 1.02033]  p=0.43654 | 0.99193 [0.94389, 1.04242]  p=0.74909 | 1.03135 [0.99505, 1.06897]  p=0.09127 |
| IL-4 | 0.86751 [0.58651, 1.28314]  p=0.47666 | 0.88864 [0.69521, 1.13590]  p=0.34588 | 0.82453 [0.63397, 1.07237]  p=0.15017 |
| TRAIL | 1.00781 [0.98602, 1.03009]  p=0.48545 | 1.00593 [0.98084, 1.03167]  p=0.64638 | 0.98986 [0.97001, 1.01012]  p=0.32433 |
| IL-9 | 1.00024 [0.99954, 1.00095]  p=0.49830 | 1.00065 [0.99968, 1.00161]  p=0.19043 | 0.99968 [0.99897, 1.00040]  p=0.38221 |
| EOTAXIN | 0.99754 [0.99045, 1.00468]  p=0.49843 | 0.99609 [0.98588, 1.00640]  p=0.45580 | 0.99208 [0.98297, 1.00129]  p=0.09161 |
| SDF-1ALFA | 0.99989 [0.99953, 1.00024]  p=0.54075 | 0.99957 [0.99923, 0.99992]  **p=0.01683** | 0.99997 [0.99953, 1.00042]  p=0.90356 |
| IL-8 | 0.97998 [0.91578, 1.04869]  p=0.55866 | 0.87059 [0.76421, 0.99177]  **p=0.03714** | 0.96250 [0.90612, 1.02239]  p=0.21457 |
| IL-10 | 1.00150 [0.99631, 1.00672]  p=0.57101 | 0.99297 [0.95987, 1.02721]  p=0.68335 | 1.01584 [0.99678, 1.03527]  p=0.10389 |
| IL-13 | 0.98626 [0.93708, 1.03803]  p=0.59608 | 0.99457 [0.94574, 1.04592]  p=0.83217 | 0.98764 [0.92154, 1.05847]  p=0.72487 |
| RANTES | 1.00000 [0.99999, 1.00002]  p=0.61354 | 1.00000 [0.99999, 1.00000]  p=0.39911 | 1.00000 [0.99999, 1.00001]  p=0.75840 |
| IL-16 | 0.99720 [0.98424, 1.01034]  p=0.67471 | 0.99619 [0.98157, 1.01103]  p=0.61292 | 1.00179 [0.99590, 1.00772]  p=0.55157 |
| IFN-GAMMA | 0.99611 [0.97653, 1.01608]  p=0.70027 | 0.99827 [0.98469, 1.01204]  p=0.80428 | 0.99255 [0.97367, 1.01180]  p=0.44557 |
| IL-1RA | 0.99916 [0.99485, 1.00350]  p=0.70426 | 1.00042 [0.99950, 1.00135]  p=0.36857 | 0.99957 [0.99735, 1.00179]  p=0.70373 |
| MCP-3 | 0.99155 [0.94429, 1.04117]  p=0.73330 | 0.92984 [0.85357, 1.01293]  p=0.09577 | 1.04452 [0.98890, 1.10326]  p=0.11871 |
| SCF | 0.99907 [0.99325, 1.00493]  p=0.75551 | 0.99188 [0.97938, 1.00455]  p=0.20796 | 1.00176 [0.99898, 1.00455]  p=0.21537 |
| IL-1ALFA | 0.99885 [0.99156, 1.00619]  p=0.75764 | 0.99680 [0.98367, 1.01010]  p=0.63501 | 1.00532 [0.99844, 1.01224]  p=0.13006 |
| IL-2RALFA | 0.99859 [0.98845, 1.00883]  p=0.78665 | 0.99920 [0.98559, 1.01299]  p=0.90850 | 0.99830 [0.98461, 1.01219]  p=0.80963 |
| MIP-1BETA | 0.99957 [0.99627, 1.00287]  p=0.79626 | 1.00156 [0.99733, 1.00581]  p=0.47130 | 0.99838 [0.99601, 1.00076]  p=0.18323 |
| IP-10 | 0.99987 [0.99889, 1.00086]  p=0.80197 | 0.99979 [0.99876, 1.00082]  p=0.69248 | 1.00011 [0.99981, 1.00041]  p=0.46192 |
| TNF-ALFA | 0.99914 [0.99151, 1.00683]  p=0.82643 | 0.99949 [0.99117, 1.00789]  p=0.90566 | 0.99996 [0.99363, 1.00633]  p=0.99050 |
| LIF | 0.99972 [0.99643, 1.00303]  p=0.86969 | 0.99752 [0.99077, 1.00432]  p=0.47422 | 1.00298 [0.99895, 1.00702]  p=0.14745 |
| TNF-BETA | 1.00020 [0.99767, 1.00273]  p=0.87878 | 1.00115 [0.99928, 1.00303]  p=0.22665 | 0.99933 [0.99777, 1.00089]  p=0.40158 |
| MIF | 1.00003 [0.99959, 1.00047]  p=0.89332 | 1.00006 [0.99991, 1.00022]  p=0.41327 | 1.00000 [0.99987, 1.00012]  p=0.96503 |
| HGF | 1.00007 [0.99837, 1.00178]  p=0.93588 | 0.99800 [0.99494, 1.00107]  p=0.20105 | 0.99948 [0.99616, 1.00281]  p=0.75999 |
| BETA-NGF | 0.99808 [0.79250, 1.25699]  p=0.98697 | 0.65629 [0.44092, 0.97687]  **p=0.03796** | 0.90181 [0.63546, 1.27981]  p=0.56282 |

^1^Relative Risk (RR), 95% Confidence Interval (95% CI)

**Table S7:** Univariable Poisson regression for pCR stratified by molecular subtype

| **Cytokine** | **HER2-enriched**^1^  n=14 (3 pCR/11 non-pCR) | **Luminal**^1^  n=38 (6 pCR/32 non-pCR) | **TNBC**^1^  n=16 (1 pCR/15 non-pCR) |
| --- | --- | --- | --- |
| FGF-BASIC | 0.95029 [0.92849, 0.97261]  **p<0.001** | 0.98966 [0.96649, 1.01339]  p=0.38985 | 0.24168 [0.21450, 0.27230]  **p<0.001** |
| EOTAXIN | 0.90068 [0.85489, 0.94892]  **p<0.001** | 0.99836 [0.97457, 1.02273]  p=0.89415 | 0.91322 [0.84717, 0.98443]  **p=0.01780** |
| MIG | 1.00032 [1.00006, 1.00058]  **p=0.01632** | 0.99886 [0.99661, 1.00110]  p=0.31750 | 0.99680 [0.99376, 0.99984]  **p=0.03932** |
| SCF | 0.92842 [0.87338, 0.98693]  **p=0.01722** | 0.97742 [0.93965, 1.01669]  p=0.25580 | 0.86959 [0.78600, 0.96206]  **p=0.00673** |
| IL-4 | 0.25160 [0.07816, 0.80989]  **p=0.02069** | 0.83013 [0.58689, 1.17418]  p=0.29263 | 1.09e-26 [6.78e-29, 1.77e-24]  **p<0.001** |
| SCGF-BETA | 0.99996 [0.99992, 0.99999]  **p=0.02220** | 1.00000 [0.99997, 1.00003]  p=0.97437 | 0.99998 [0.99996, 1.00000]  **p=0.04784** |
| IL-18 | 0.91843 [0.84971, 0.99271]  **p=0.03201** | 1.01302 [0.98600, 1.04079]  p=0.34831 | 0.92573 [0.86752, 0.98784]  **p=0.01985** |
| MIP-1BETA | 0.98823 [0.97698, 0.99960]  **p=0.04256** | 1.00834 [0.99874, 1.01803]  p=0.08886 | 0.98100 [0.97045, 0.99165]  **p<0.001** |
| TNF-BETA | 0.99062 [0.98090, 1.00043]  p=0.06081 | 1.00371 [0.99871, 1.00873]  p=0.14600 | 0.98333 [0.97412, 0.99262]  **p<0.001** |
| IL-17A | 0.62489 [0.38133, 1.02402]  p=0.06207 | 1.09392 [1.02982, 1.16202]  **p=0.00357** | 0.46542 [0.21883, 0.98987]  **p=0.04698** |
| IL-9 | 0.99433 [0.98841, 1.00029]  p=0.06229 | 1.00221 [0.99998, 1.00446]  p=0.05252 | 0.98784 [0.98005, 0.99569]  **p=0.00244** |
| SDF-1ALFA | 0.99868 [0.99722, 1.00014]  p=0.07680 | 0.99959 [0.99869, 1.00049]  p=0.37158 | 1.00014 [0.99936, 1.00092]  p=0.72731 |
| TNF-ALFA | 0.97852 [0.95006, 1.00782]  p=0.14911 | 1.00133 [0.97501, 1.02835]  p=0.92225 | 1.28e-05 [4.10e-06, 4.00e-05]  **p<0.001** |
| PDGF-BB | 0.97244 [0.93590, 1.01040]  p=0.15258 | 0.99985 [0.99955, 1.00016]  p=0.34826 | 0.96503 [0.93458, 0.99646]  **p=0.02951** |
| M-CSF | 0.91646 [0.81220, 1.03411]  p=0.15687 | 0.97728 [0.82636, 1.15575]  p=0.78826 | 0.95959 [0.86575, 1.06359]  p=0.43201 |
| IL-8 | 1.08880 [0.96032, 1.23447]  p=0.18416 | 0.90713 [0.73188, 1.12435]  p=0.37351 | 4.30e-13 [2.83e-14, 6.53e-12]  **p<0.001** |
| IL-2RALFA | 0.98558 [0.96376, 1.00790]  p=0.20372 | 1.01457 [0.97786, 1.05267]  p=0.44166 | 0.03182 [0.02361, 0.04288]  **p<0.001** |
| LIF | 0.99074 [0.97657, 1.00512]  p=0.20583 | 0.99217 [0.98097, 1.00350]  p=0.17484 | 1.00419 [0.99273, 1.01579]  p=0.47515 |
| IL-13 | 0.90208 [0.76361, 1.06565]  p=0.22545 | 0.99575 [0.88278, 1.12318]  p=0.94472 | 0.76944 [0.63187, 0.93697]  **p=0.00911** |
| CTACK | 0.99488 [0.98625, 1.00359]  p=0.24878 | 1.00024 [0.99645, 1.00403]  p=0.90235 | 0.99926 [0.99623, 1.00229]  p=0.63022 |
| IP-10 | 1.00145 [0.99897, 1.00394]  p=0.25106 | 0.99942 [0.99704, 1.00180]  p=0.63059 | 0.99856 [0.99695, 1.00017]  p=0.07915 |
| MIF | 0.99815 [0.99498, 1.00132]  p=0.25153 | 1.00032 [1.00005, 1.00059]  **p=0.01959** | 0.99180 [0.98568, 0.99796]  **p=0.00910** |
| IL-1BETA | 0.95590 [0.87860, 1.04001]  p=0.29456 | 0.94498 [0.85529, 1.04407]  p=0.26598 | 1.06928 [0.96999, 1.17874]  p=0.17790 |
| MCP-3 | 0.88106 [0.69245, 1.12103]  p=0.30284 | 0.88260 [0.73640, 1.05784]  p=0.17654 | 0.89481 [0.73442, 1.09021]  p=0.27006 |
| HGF | 0.99573 [0.98758, 1.00394]  p=0.30729 | 1.00233 [0.99551, 1.00920]  p=0.50391 | 0.24546 [0.21492, 0.28034]  **p<0.001** |
| GRO-ALFA | 0.99910 [0.99736, 1.00084]  p=0.30932 | 1.00073 [0.99950, 1.00197]  p=0.24534 | 0.96454 [0.96073, 0.96836]  **p<0.001** |
| IFN-GAMMA | 0.95678 [0.87352, 1.04797]  p=0.34146 | 1.00880 [0.97572, 1.04301]  p=0.60643 | 0.00386 [0.00230, 0.00647]  **p<0.001** |
| RANTES | 0.99993 [0.99977, 1.00008]  p=0.34829 | 1.00000 [0.99999, 1.00000]  p=0.20336 | 0.99970 [0.99950, 0.99990]  **p=0.00290** |
| IL-10 | 0.99047 [0.96749, 1.01400]  p=0.42410 | 0.98364 [0.94389, 1.02507]  p=0.43319 | 1.03267 [0.97610, 1.09253]  p=0.26337 |
| IL-6 | 0.88104 [0.63929, 1.21422]  p=0.43898 | 0.92237 [0.65320, 1.30246]  p=0.64623 | 1.47e-78 [2.68e-86, 8.02e-71]  **p<0.001** |
| IL-3 | 0.22249 [0.00474, 10.44015]  p=0.44404 | 0.08694 [0.00741, 1.02004]  p=0.05187 | 1.62e-61 [3.01e-123, 8.68615]  p=0.05358 |
| MCP-1-MCAF | 1.02885 [0.94251, 1.12309]  p=0.52481 | 0.99525 [0.98498, 1.00563]  p=0.36820 | 3.56e-06 [1.08e-06, 1.17e-05]  **p<0.001** |
| IL-5 | 0.99783 [0.99104, 1.00468]  p=0.53388 | 0.99997 [0.99224, 1.00775]  p=0.99339 | 0.56750 [0.53259, 0.60469]  **p<0.001** |
| IL-1RA | 1.00311 [0.99120, 1.01515]  p=0.61050 | 1.00202 [1.00021, 1.00384]  **p=0.02847** | 0.99490 [0.99013, 0.99969]  **p=0.03702** |
| IL-1ALFA | 0.99317 [0.96594, 1.02116]  p=0.62886 | 0.98508 [0.95622, 1.01480]  p=0.32151 | 1.01612 [0.99148, 1.04137]  p=0.20173 |
| MIP-1ALFA | 1.22625 [0.19811, 7.59015]  p=0.82641 | 1.33237 [0.55906, 3.17534]  p=0.51723 | 1.26e-47 [1.20e-51, 1.32e-43]  **p<0.001** |
| IL-12-P40 | 0.99789 [0.97892, 1.01723]  p=0.82956 | 1.00124 [0.97945, 1.02352]  p=0.91187 | Not converged |
| IL-16 | 0.99684 [0.96387, 1.03093]  p=0.85360 | 0.99558 [0.97372, 1.01793]  p=0.69585 | 0.34224 [0.32209, 0.36365]  **p<0.001** |
| BETA-NGF | 0.91236 [0.32098, 2.59325]  p=0.86336 | 0.84045 [0.35803, 1.97287]  p=0.68971 | 0.34131 [0.14865, 0.78369]  **p=0.01125** |
| G-CSF | 0.99815 [0.96894, 1.02823]  p=0.90255 | 1.00199 [0.96190, 1.04375]  p=0.92407 | 0.82270 [0.71015, 0.95309]  **p=0.00932** |
| TRAIL | 1.00611 [0.90606, 1.11721]  p=0.90924 | 1.02059 [0.95684, 1.08859]  p=0.53567 | 0.83426 [0.71649, 0.97137]  **p=0.01959** |
| VEGF-A | 0.99940 [0.98548, 1.01353]  p=0.93376 | 1.01966 [0.98709, 1.05330]  p=0.23980 | 0.52875 [0.49093, 0.56948]  **p<0.001** |

^1^Relative Risk (RR), 95% Confidence Interval (95% CI)
